# Molecular Modeling of the *Shigella flexneri* Serogroup 3 and 5 O-Antigens and Conformational Relationships for a Vaccine Containing Serotypes 2a and 3a

**DOI:** 10.3390/vaccines8040643

**Published:** 2020-11-02

**Authors:** Jason Hlozek, Sara Owen, Neil Ravenscroft, Michelle M. Kuttel

**Affiliations:** 1 Department of Chemistry, University of Cape Town, Rondebosch 7701, South Africa; jason.hlozek@live.co.za (J.H.); neil.ravenscroft@uct.ac.za (N.R.); 2 Department of Computer Science, University of Cape Town, Rondebosch 7701, South Africa; saraowen99@gmail.com

**Keywords:** O-antigen, conformation, *Shigella flexneri*, molecular modelling, cross protection

## Abstract

The pathogenic bacterium *Shigella flexneri* is a leading global cause of diarrheal disease. The O-antigen is the primary vaccine target and distinguishes the 30 serotypes reported. Except for serotype 6, all *S. flexneri* serotypes have a common backbone repeating unit (serotype Y), with variations in substitution creating the various serotypes. A quadrivalent vaccine containing serotypes 2a and 3a (as well as 6 and *Shigella sonnei*) is proposed to provide broad protection against non-vaccine *S. flexneri* serotypes through shared epitopes and conformations. Here we model the O-antigen (O-Ag) conformations of serogroups 3 and 5: a continuation of our ongoing systematic study of the *S. flexneri* O-antigens that began with serogroup 2. Our simulations show that *S. flexneri* serogroups 2, 3, and 5 all have flexible O-Ags, with substitutions of the backbone altering the chain conformations in different ways. Our analysis suggests three general heuristics for the effects of substitution on the *Shigella* O-Ag conformations: (1) substitution on rhamnose C reduces the extension of the O-Ag chain; (2) substitution at O-3 of rhamnose A restricts the O-Ags to predominantly helical conformations, (3) substitution at O-3 of rhamnose B has only a slight effect on conformation. The common O-Ag conformations across serotypes identified in this work support the assumption that a quadrivalent vaccine containing serotypes 2a and 3a could provide coverage against *S. flexneri* serotype 3b and serogroup 5.

## 1. Introduction

Diarrheal diseases cause over 1.6 million deaths each year [1], disproportionately affecting low-income regions [2] and young children [3]. *Shigella flexneri* is a leading cause of enteric infections, with no licensed vaccine currently available [4]. The increasing prevalence of antibiotic resistant strains [5,6,7,8] necessitates a broad coverage *Shigella* vaccine to prevent infection [4,9] and reduce the global disease burden [10,11].

**Figure 1:**
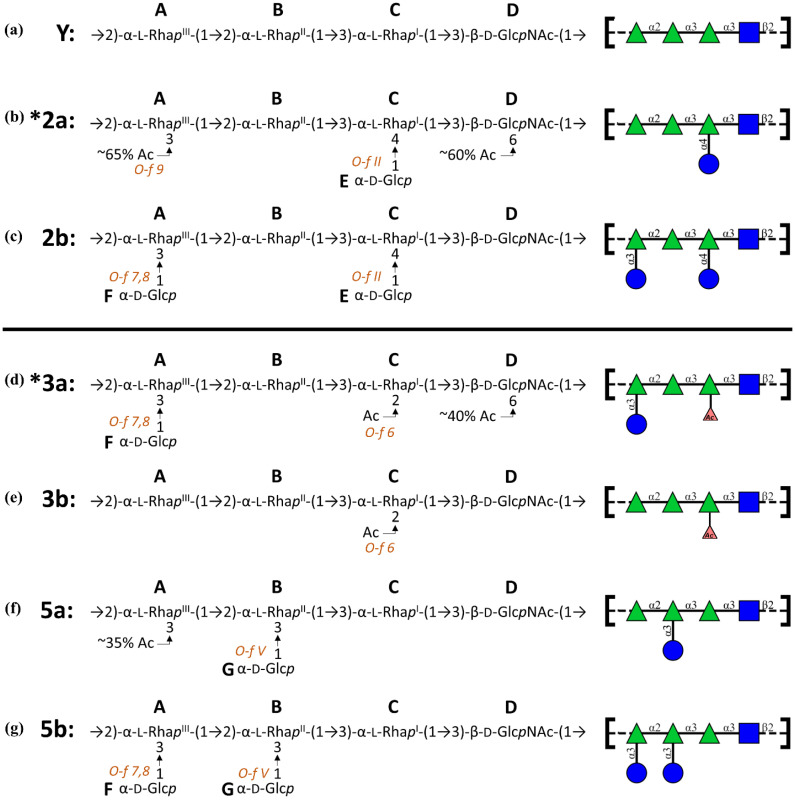
Line structures and diagrams of the *Shigella flexneri* O-antigen repeating units of serotypes (**a**) Y, (**b**) 2a, (**c**) 2b, (**d**) 3a, (**e**) 3b, (**f**) 5a, and (**g**) 5b. All serogroups share the serotype Y backbone and are distinguished by substitutions, which are labelled with the associated O-factors. Vaccine serotypes 2a and 3a are indicated with an asterisk. Schematic diagrams are depicted using the Symbol Nomenclature for Glycans (SNFG) symbol set [35] where green triangle—Rha, blue square—GlcNAc, blue circle—Glc, red triangle—O-acetylation.

The structure of the *S. flexneri* O-antigen (O-Ag) repeating unit (RU)—the carbohydrate component of the cell-surface lipopolysaccharide—classifies strains into approximately 30 serotypes and seven serogroups [12,13]. The O-Ag is the primary target of the host immune response [14,15] and is a focus of current vaccine development [16,17]. Except for serotype 6, all *S. flexneri* serotypes have the same backbone RU-serotype Y: →2)-α-L-Rha*p*^III^-(1→2)-α-L-Rha*p*^II^-(1→ 3)-α-L-Rha*p*^I^-(1→3)-β-D-Glc*p*NAc-(1→ (Figure 1a). Serotypes are defined by the combination of type O-factor epitopes in the O-Ag RU (which distinguish the serogroups from each other) as well as the group O-factors determined by glucosylation, O-acetylation, and phosphorylation substitution of the serotype Y backbone, which appear across serogroups [13,18]. The similarities between *S. flexneri* O-Ags (shared backbone and group O-factors) suggest that some serotypes may cross-protect, enabling development of a broad-coverage vaccine with minimal valency. The proposed vaccine for *Shigella* based on the Global Enteric Multicenter Study (GEMS) consists of *S. flexneri* serotypes 2a, 3a, 6, and the single *S. sonnei* serotype, which is estimated to provide direct protection against 64% of *Shigella* strains causing infection in children in low-income areas, with cross-reactivity potentially extending this up to 88% [19,20,21]. Serotypes 2a and 3a were chosen as vaccine components because both are prevalent causes of infection (ranked 1st and 4th, respectively) and together they express the group O-factor epitopes (group O-factors 6; 7,8; 9) found on most remaining non-vaccine serotypes, allowing for potential broad cross-protection.

As cross-protection between antigens is expected to require both chemical and conformational similarity [22], molecular modeling can provide insight into the potential for cross-protection between *S. flexneri* O-Ags. Early computational models indicated an extended conformation for serotype Y [23], and a helix for serotype 5a [24]. However, MD simulations predicted that serotype Y is highly flexible [25,26] and that 3 RU of 12 *S. flexneri* O-Ags show similar conformations to each other [27,28]. Although *S. flexneri* expresses a heterogenous distribution of O-Ag chain lengths, 3 RU is considered sufficient to represent the O-antigen conformation [17,27].

We previously embarked on a systematic conformational study of all the *Shigella* O-Ags, beginning with the backbone (serotype Y) and the serogroup 2 O-Ags [26]. For the serotype 2a O-Ag (Figure 1b), we found that glucosylation on O-4 of rhamnose C (type O-factor II) [13] restricted the O-Ag to more compact conformations as compared to the highly flexible, unsubstituted serotype Y. Additional substitution on O-3 of rhamnose A produced more extended helical conformations, regardless of whether it was O-acetylation (serotype 2a, group O-factor 9) or glucosylation (serotype 2b; group O-factor 7,8; Figure 1c). This work indicated that an O-3-acetylated 2a O-Ag (expressing group O-factor 9) may provide stronger cross-protection against 2b (ranked 3rd in prevalence, expressing group O-factor 7,8) than 2a (which lacks both O-factors) and may provide enhanced coverage of other serotypes expressing O-factor 9 (serotypes Y_1_, 1a_1_, 1b, 5a_1_, 6, and 7a_1_) [13].

Here we report the next step of our study, modeling the conformations of serotypes 3a, 3b, and serogroup 5. Serotype 3a is a component of the proposed quadrivalent vaccine [19,20] as it is prevalent globally [19]. Serotype 3b is not included in the vaccine, but is prevalent in parts of Asia [29,30]. In contrast, serogroup 5 has a relatively low incidence of disease; serotype 5a has been widely studied and is used as a reference strain, whereas 5b accounted for some cases in the GEMS report [19].

All serotypes in serogroup 3 contain O-acetylation on O-2 of rhamnose C (group O-factor 6) [13], which is important for antibody recognition [31,32]. Serotype 3a (Figure 1d) is defined by additional glucosylation on O-3 of rhamnose A (O-factor 7,8), with subtype 3a_1_ having partial O-acetylation on O-6 of GlcNAc D (O-factor 10, ≈40%); neither of which are present in serotype 3b (Figure 1e) [12,13]. Serotype 5a (Figure 1f) is defined by glucosylation at position O-3 of rhamnose B (type O-factor V) with subtype 5a_1_ having partial O-3-acetylation on rhamnose A (group O-factor 9, ≈35%) [13]. The most common 5a strain for laboratory study (M90T) has no O-acetylation [33,34]. Serotype 5b (Figure 1g) is glucosylated on O-3 of rhamnose A (O-factor 7,8) with no O-acetylation.

Some studies have indicated cross-reactivity between serogroups 2, 3, and 5. Serotypes 2a, 3b, and 5a each react with group O-factor 3,4 antisera (associated backbone trisaccharide residues C-D-A) [12] and partial cross-reactivity with these strains is demonstrated from serotype 2a in human testing [36]. Serotypes 2b, 3a, and 5b share glucosylation on O-3 of rhamnose A (group O-factor 7,8) and strong cross-reactivity is reported from a candidate 2a/3a vaccine against 2b and 5b in guinea pigs [37]. Therefore, a vaccine containing serotypes 2a and 3a (expressing group O-factors 6; 7,8 and 9) is suggested to elicit broad cross-protection against the remaining serogroup 2, 3, and 5 serotypes [19].

Here we compare simulations of serogroups 3 and 5 with our recent work on the serogroup 2 O-Ags, contrasting the O-Ag behavior for O-acetylated serotype 2a (group O-factor 9) and 3a (group O-factors 6 and 7,8) with the non-vaccine serotype 3b (group O-factor 6) as well as serotypes 5a (type O-factor V) and 5b (group O-factor 7,8). With this large data set for comparison, we aim to broadly identify guiding heuristics for the conformational effect of substitutions on particular positions of the *Shigella* backbone, specifically substitutions on rhamnose C (O-factor II in serogroup 2 and O-factor 6 in serogroup 3); O-3 of rhamnose A (O-factor 9 in serotype 2a-3Ac; O-factor 7,8 in serotypes 2b, 3a, and 5b); O-3 of rhamnose B (O-factor V in serogroup 5). We assume that O-factors with a significant conformational effect should be represented in the vaccine serotypes to allow for broad coverage. Ultimately, we aim to determine whether the conformational findings from our computational modeling supports the assumption that a quadrivalent vaccine containing *S. flexneri* serotypes 2a and 3a (as well as 6 and *S. sonnei*) could provide broad coverage against *S. flexneri* serotype 3b and serogroup 5.

## 2. Materials and Methods

The *S. flexneri* O-Ags have glycosidic linkages described by two dihedral angles, φ and ψ, defined as φ = H1-C1-O1-C_x_’ and ψ = C1-O1-C_x_’-H_x_’. These definitions are analogous to φ_H_ and ψ_H_ in IUPAC nomenclature and are consistent with our previous carbohydrate modeling [26,38] This work follows our established methodology for the computational study of carbohydrate antigens. Potential of mean force (PMF) calculations for the disaccharide fragments of the O-Ag repeating unit indicate the global minima for each linkage, which are then used to build short 3 RU chains for initial 300 ns MD simulations in solution. The most populated linkage conformations from the 3 RU simulations are then used to construct starting structures for the simulations of the 6 RU chains [39,40,41].

### 2.1. φ, ψ PMF Calculations

The low-energy conformations of the glycosidic linkages were determined by calculating the potential of mean force (PMF) for rotation about the φ and ψ dihedral angles of each disaccharide linkage. PMFs were calculated with the metadynamics algorithm [42] as implemented in NAMD [43]. The disaccharide PMFs were calculated in the gas-phase with the φ, ψ dihedral angles set as collective variables.

### 2.2. Molecular Dynamics

Simulations were run with the NAMD software package [43], employing CUDA extensions to leverage graphics processing units for the calculation of long-range electrostatic potentials and non-bonded forces [44]. Carbohydrates were modelled with the CHARMM36 additive force field for carbohydrates [45,46] and explicit water molecules were represented with the TIP3P water model [47].

Our in-house CarbBuilder software was used to build the carbohydrate structures prior to simulation [48]. Initial 3 RU chains of the serogroup 3 and 5 O-Ags (not discussed here) were built with glycosidic linkage conformations set to the energy minimum of the respective disaccharide PMFs. The RUs of the O-Ag chains modeled in this study are as follows with the serotype-defining moieties in bold:

3a: →2)-*[αDGlc(1→3)]*αLRha(1→2)-αLRha(1→3)-αLRha2Ac(1→3)*-*βDGlcNAc-(1→
3b: →2)-αLRha(1→2)-αLRha(1→3)-αLRha2Ac(1→3)*-*βDGlcNAc-(1→
5a: →2)-αLRha(1→2)-*[αDGlc(1→3)]*αLRha(1→3)-αLRha(1→3)*-*βDGlcNAc-(1→
5b: →2)-*[αDGlc(1→3)]*αLRha(1→2)-*[αDGlc(1→3)]*αLRha(1→3)-αLRha(1→3)*-*βDGlcNAc-(1→

The 3 RU simulations were run for 1000 and 300 ns (for serogroups 3 and 5, respectively), with the most frequent dihedral angles from these simulations used to build the initial conformations for the 6 RU chains. These conformations were then subjected to 10,000 steps of standard NAMD minimization in vacuum and subsequently placed into a cubic water box with the *solvate* command from the Visual Molecular Dynamics (VMD) package [49]. The cubic water boxes for the 6 RU structures had side lengths of 100 and 90 Å, respectively, for serogroups 3 and 5, and periodic boundary conditions were employed. The solvated structures were gradually heated through a protocol of 5 K incremental temperature reassignments between 10 and 310 K, with 1000 steps of NAMD minimization and 1000 steps of MD after each temperature reassignment.

Equations of motion were integrated using the velocity-Verlet method [50] with a 1 fs step size. Molecular dynamics simulations were performed under the isothermal-isobaric (nPT) ensemble at a temperature of 310 K and maintained with a Langevin piston barostat [43] and Nose-Hoover thermostat—a hybridized method of the Nose-Hoover constant pressure method [51] with piston fluctuations controlled by Langevin dynamics [52], as implemented in NAMD. Particle mesh Ewald (PME) summation [53] was used for calculation of long-range electrostatics, with k = 0.20 Å^−1^ and PME grid dimensions that were set equal to the periodic cell size. Non-bonded interactions were truncated at 15.0 Å and a switching function implemented between 12.0 and 15.0 Å. The 1–4 interactions were not scaled, in accordance with CHARMM force field recommendations.

### 2.3. Block Averaging Analysis

Block averaging analysis is used to assess simulation convergence and is implemented with in-house Python scripts [54]. The block averaging analysis algorithm splits a simulation trajectory with *N* frames into a set of *M* “blocks” with a length of *n* frames, such that *N* = *M* × *n*. Next, an average of a selected measurable (e.g., end-to-end distance) is calculated within each block. The block length (*n*) is slowly increased and, at each value of *n*, the set of block averages are recalculated. The standard deviation in the set of block averages, σ*_n_*, is used to determine the blocked standard error (BSE) for each value of *n*. The simulation is indicated to be converged once the running estimate of the BSE asymptotes to a plateau, where the plateau represents the true standard error in the estimate of the mean [55].

### 2.4. Data Analysis

Simulations underwent 200 ns of equilibration followed by production runs of 1 and 2 µs (for serogroups 3 and 5, respectively). Snapshots of molecular conformations were taken at 25 ps intervals from the simulation trajectories. Inter-atomic distances and dihedral angles were measured from VMD’s Tcl console and graphical user interface, and statistical calculations were performed with in-house Python scripts. For all saccharides, we defined the end-to-end distance, *r*, as the length from C-2 of rhamnose B at the non-reducing end of the chain and C-1 of rhamnose C at the reducing end, thus excluding the very flexible terminal sugar units.

Molecular conformations were visualized in VMD [49], with carbohydrate rings highlighted by the PaperChain visualization algorithm [56]. Before conformational clustering, the trajectory snapshots were aligned on the ring atoms of the central ‘C-D-A-B’ fragment between RU3 and RU4—a frame-shifted full repeating unit to account for the variability in each linkage across the different serotypes. The most common chain conformations are determined by clustering the simulation snapshots into families with relative occupancies. We cluster the central 4 RU of each 6 RU chain, as the terminal repeating units are less representative of the native O-Ag backbones. VMD’s internal *cluster* command was employed to calculate the conformational clusters in the production runs with an RMSD fit of the non-hydrogen atoms in the central 4 RUs with a cut-off of 5.5 Å. Clusters comprising less than 5% of the simulation were excluded. The conformations of the previously published serotype Y and serogroup 2 simulations [26] were recalculated under these criteria for a fair comparison between the serogroups.

## 3. Results

We begin our analysis of the simulation data with a broad comparison of the O-Ag chain extension and flexibility of *S. flexneri* serogroup 2 with serogroups 3 and 5; then we analyze the dominant backbone conformation of each O-Ag; finally, we explore the conformational effects of the glucosylation and O-acetylation on the orientations of the backbone glycosidic linkages.

### 3.1. Simulation Convergence

We used block averaging analysis [54,55] of two metrics of chain flexibility—the end-to-end distance, *r*, and the radius of gyration, R_g_—to assess the convergence of the MD simulations. Convergence is indicated by the plots of the blocked standard error (BSE) for both r and R_g_ (shown in Figure S1a,b) reaching a plateau. The asymptote of the BSE plot represents the true standard error in the measured variable, which can be used to approximate a correlation time for the simulation. The range of correlation times from 18 to 104 ns indicate that the 200 ns equilibration period is sufficient for all the O-Ags in this study. Further analysis reveals that the number of statistically independent samples in each simulation is much greater than 1 (49, 58, 88, and 21 for 3a, 3b, 5a, and 5b, respectively)—as recommended for a converged trajectory [55]. Therefore, block averaging analysis indicates that the longer production runs (1000 ns for serogroup 3 and 2000 ns for serogroup 5) provide sufficient sampling of the conformational space. The simulations of the more flexible serogroup 5 O-Ags were extended to 2 µs to ensure that convergence was achieved.

### 3.2. O-Ag Flexibility

**Figure 2:**
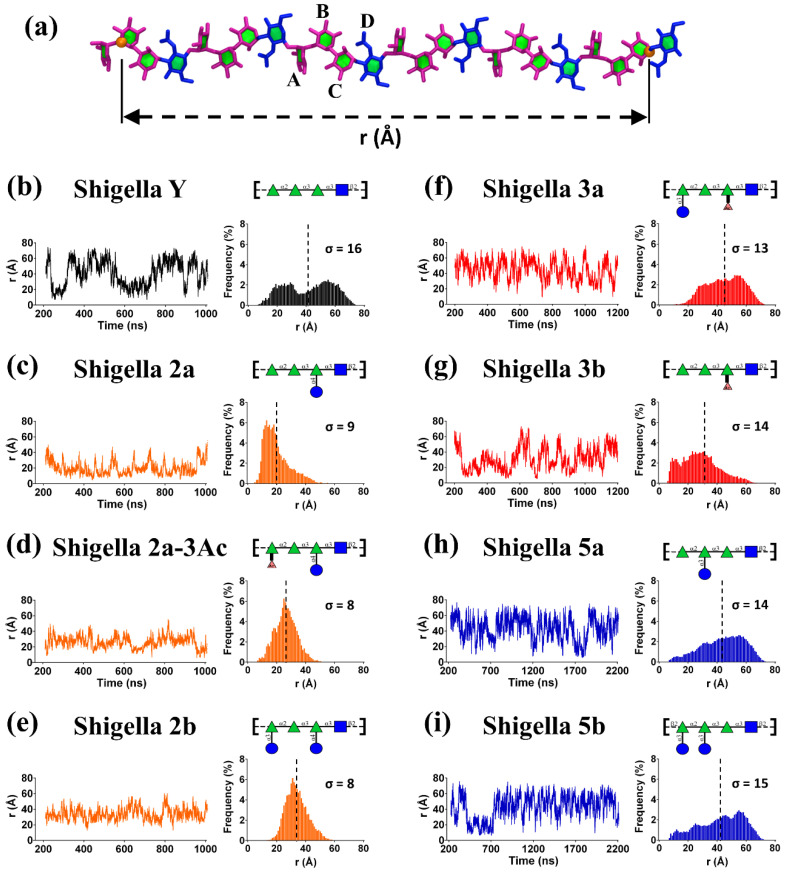
Comparison of the *r* time series and corresponding histograms for 6 RU simulations of the modeled *S. flexneri* O-Ags. (**a**) A 6 RU model of the serotype Y O-Ag depicted with the end-to-end distance, *r*; rhamnose is colored pink and N-acetyl-glucosamine blue. The *r* time series (left column for each serotype) and corresponding distribution (right column for each serotype) are shown for (**b**) Y, (**c**) 2a, (**d**) O-3 acetylated 2a, (**e**) 2b, (**f**) 3a, (**g**) 3b, (**h**) 5a, and (**i**) 5b. The mean for each histogram distribution is depicted with a dashed line and the corresponding standard deviation is indicated.

The fluctuation in *r* over the course of a simulation is a simple measure of molecular extension and flexibility for the *S. flexneri* O-Ags. Here we define *r* as the distance between C-2 of rhamnose B in RU1 and C-1 of rhamnose C in RU6 (Figure 2a). Figure 2 compares the *r* time series and corresponding histograms for the simulations of the serotype Y backbone and the serogroup 2 O-Ags previously published [26] with simulations of serogroup 3 (Figure 2f,g) and serogroup 5 (Figure 2h,i).

For a first broad comparison of the O-Ags, a scan of the graphs in Figure 2 quickly reveals that the unsubstituted Y backbone (Figure 2b) is by far the most flexible of the O-Ags, showing the greatest range of *r* values (σ ≈ 16 Å), whereas the serogroup 2 O-Ags (Figure 2c–e) are the least flexible (σ ≈ 8–9 Å). Serogroups 3 and 5 fall between these two extremes, with serogroup 3 being somewhat less flexible (σ ≈ 13 Å for 3a, 14 Å for 3b) than serogroup 5 (σ ≈ 14 Å for 5a, 15 Å for 5b). Moreover, the distributions of *r* vary considerably across the O-Ags. The flexible serogroup Y has a bimodal distribution, in stark contrast to the well-defined tight distributions shown for serogroup 2.

The graphs of *r* reveal that the effect of glucosylation on the conformation and dynamics of the O-Ag chain depends on the glucosylation position: compare the graph of serotype 2a expressing O-factor II (O-4 glucosylation on rhamnose C, Figure 2c) with 5a expressing O-factor V (O-3 glucosylation on rhamnose B, Figure 2h). In particular, the O-factor II glucosylation that defines serogroup 2 has a very dramatic effect on *r*, reducing the overall extension and flexibility of the chain. In contrast, the O-factor V glucosylation that defines serogroup 5 has a less obvious effect on *r*, slightly increasing the average chain extension. Finally, O-factor 7,8 (O-3 glucosylation on rhamnose A; serotypes 2b, 3a, and 5b) appears to narrow the distribution of *r*, making the O-Ag chains more conformationally defined. For example, there is a clear difference in the shape of the *r* distribution for serotype 3a (Figure 2f) as compared to 3b (Figure 2g): serotype 3a has a unimodal distribution of r (mean 45 Å, σ ≈ 13 Å) whereas in 3b (which lacks O-factor 7,8) *r* is shifted to smaller values and has a right-skewed distribution with a peak at 25 Å (mean 29 Å, σ ≈ 14 Å). This significant shift in *r* distribution within serogroup 3 indicates a substantial conformational difference between the 3a and 3b O-Ags, and hence a significant conformational effect for O-factor 7,8. We have previously observed the same effect in serogroup 2 and it can be observed within serogroup 5, which is the least conformationally defined of the four serogroups. Serogroup 5 is the most similar to the Y backbone, but does not show the same clear bimodal distribution. The *r* distributions for serotype 5b (expressing O-factor 7,8) show a slight shift to more extended conformations (Figure 2i) as compared to 5a (Figure 2h). However, the similarity of the *r* distributions for 5a and 5b suggest similar conformational behavior for both serotypes.

Further, a comparison of the *r* histograms can reveal the general effects of O-acetylation on the O-Ag backbone. We have previously observed that O-factor 9 (O-acetylation at O-3 of rhamnose A, serotype 2a-3Ac) has a similar conformational effect to glucosylation at this position (O-factor 7,8, serotype 2b), reducing the range of *r*. The range restriction for O-acetylation is not as dramatic as with glucosylation, which is expected from the relative size of the substituents (Ac = 43 g/mol and Glc = 179 g/mol). More surprisingly, we now see that O-factor 6 (O-acetylation at O-2 of rhamnose C, serogroup 3) has a similar effect restrictive effect on *r,* as can be seen in a comparison of serotype 3b (Figure 2g) with the backbone serotype Y (Figure 2b).

A general comparison of the *r* histograms suggests three broad heuristics for the effects of substitution on the backbone (serotype Y) conformation of the *Shigella* O-Ags. First, substitution at rhamnose C (O-factor II in serogroup 2 and O-factor 6 in serogroup 3) has the most impact in reducing the flexibility and extension of the O-Ag chain, with glucosylation (2a) having a greater impact than O-acetylation (3b). The impact of substitution on rhamnose C on O-Ag conformation is supported by the fact that O-factor II defines serogroup 2 and O-factor 6 is present in all of serogroup 3. Second, any substitution on O-3 of rhamnose A (O-factor 9 in serotype 2a-3Ac; O-factor 7,8 in serotypes 2b, 3a, and 5b) shifts the O-Ag to more extended conformations. Third, O-factor V (substitution at O-3 of rhamnose B, serogroup 5) does not have a significant effect on chain conformation. Our analysis suggests that, at a first approximation, these three heuristics are additive. To test and refine these rules of thumb, as well as investigate the potential for cross-reactivity between serotypes, we now perform a detailed comparison of the chain conformations for all O-Ags.

### 3.3. O-Ag Conformations

**Figure 3:**
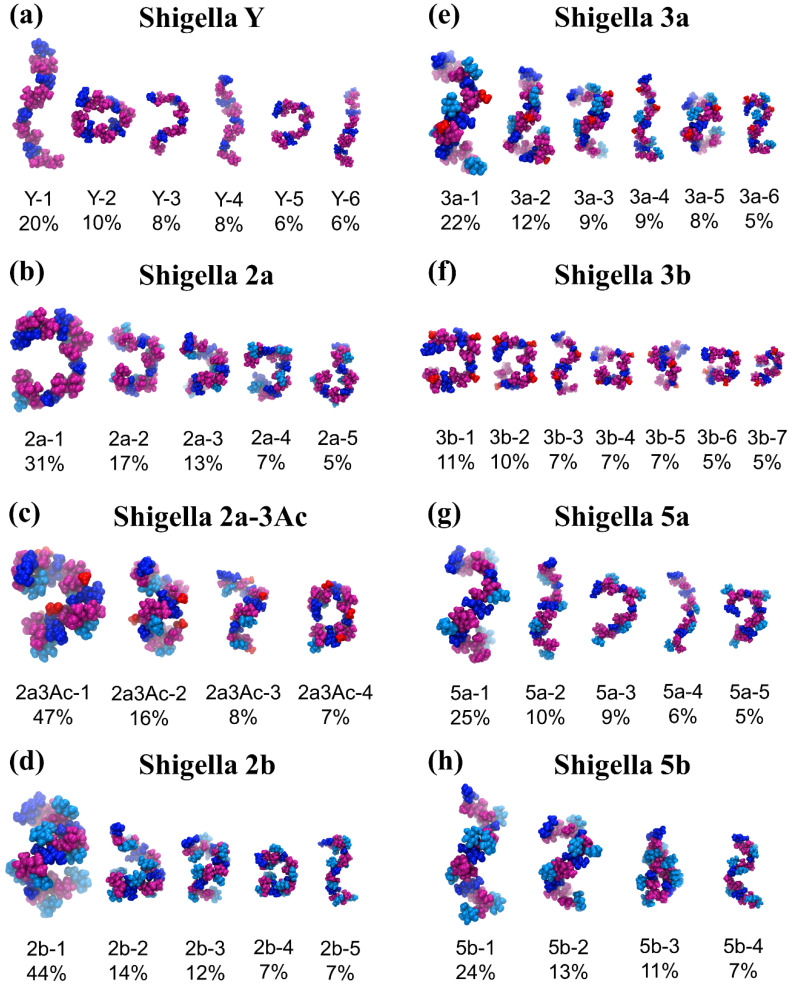
Conformational families of the central 4 RU of the 6 RU chains for (**a**) Y, (**b**) 2a, (**c**) O-3-acetylated 2a, (**d**) 2b, (**e**) 3a, (**f**) 3b, (**g**) 5a, and (**h**) 5b. Relative occupancies in the simulations (excluding the initial 200 ns) are indicated as percentages. Clusters of less than 5% are not shown. The sugars are colored: pink for Rha, dark-blue for GlcNAc, cyan for Glc side chains, and red for O-acetyl groups.

**Figure 4:**
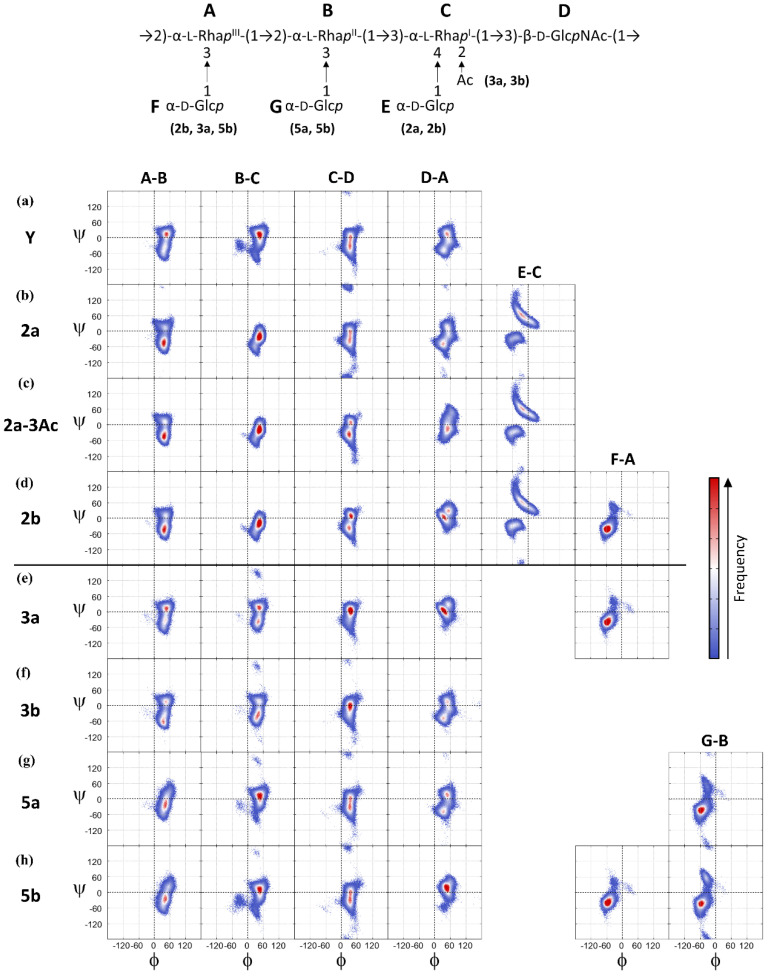
Heat map representations of scatter plots for the φ, ψ dihedrals of each glycosidic linkage for serotypes (**a**) Y, (**b**) 2a, (**c**) 2a-3Ac, (**d**) compared to serotypes (**e**) 3a, (**f**) 3b, (**g**) 5a, and (**h**) 5b in this study. The heat maps combine the points from both central repeating units (RU 3 and RU 4) to broadly sample backbone behavior. The color scale to the right indicates the relative occupancy of the dihedral angles during the simulations.

Figure 3 compares the conformational families for serotype Y (Figure 3a) and the serogroup 2 O-Ags (Figure 3b–d) with the serogroup 3 (Figure 3e,f) and serogroup 5 (Figure 3g,h) O-Ags. As discussed in prior work [26], the flexible Y backbone transitions between extended conformations (Figure 3a Y-1, Y-4, and Y-6) to more curved arrangements of the chain (Figure 3a Y-2, Y-3, and Y-5). Further, we showed that O-factor II (O-4 glucosylation at rhamnose C, serogroup 2) has a dramatic effect on the chain conformations, removing the extended conformations and restricting the O-Ag to a wide range of “C-curves” (Figure 3b). Additional substitution on O-3 of rhamnose A, whether O-acetylation in 2a (O-factor 9) or glucosylation in 2b (O-factor 7,8), was then shown to further restrict the chain and induce helical conformations (compare Figure 3c,d).

In common with serogroup 2, serogroup 3 is also substituted at rhamnose C, albeit with a smaller O-acetyl group at position 2 (O-factor 6). A comparison of the dominant conformations of 2a (Figure 3b) and 3b (Figure 3f) reveals that O-factors 6 and II have a similar effect on the backbone. For the 3b O-Ag, O-2-acetylation on rhamnose C removes most of the extended conformations of the chain and restricts the O-Ag to a range of C-curve conformations (3b-1) and other folded conformations of the chain (3b-2, 3b-4 to 3b-7). The dominant curved chain conformation for 2a (2a-1, 31%) and 3b (3b-1, 11%) are remarkably similar, indicating a similar conformational effect of substitution at rhamnose C. However, the smaller O-acetyl substituent means that the 3b chain remains more flexible than the 2a and has a minor helical conformational family (3b-3, 7%).

As for serotype 2b, serotype 3a is substituted at rhamnose C as well as rhamnose A (O-factor 7,8). This combination of substitutions has a similar effect on the conformation of the 3a O-Ag (Figure 3e) as it does on 2b (Figure 3d), producing a dominant helical conformation with 3 RU per turn and an average pitch of 29 Å (3a-1, 11%). However, 3a remains more flexible than 2b and can adopt a wide range of helical conformations (3a-3, 3a-5), as well as partially extended chains (3a-2), fully extended chains (3a-4), and S-bends (3a-6). Therefore, the serogroup 3 O-Ags follow a similar trend to serogroup 2 [26]: substitution on rhamnose C restricts the chains to curved conformations for both serotypes 2a and 3b (Figure 3b,f) and additional substitution on O-3 of rhamnose A shifts the conformations towards helices for serotypes 2a-3Ac, 2b, and 3a (Figure 3c–e). Further, the axially orientated O-acetyl groups of serogroup 3 are readily accessible for antibody binding in both serogroup 3 O-Ags, which supports the reported immunodominance of O-factor 6 [31].

A comparison of the primary conformations of serogroup 5 with the backbone shows the effect of O-factor V (glucosylation at O-3 of rhamnose B) on the chain conformation. Relative to the backbone (Figure 3a), the 5a O-Ag shows an increase in the prevalence of elongated helices (Figure 3g): the dominant conformation (5a-1, 25%) is a right-handed helix with 3 RU per turn, in agreement with an early helical model prediction [24]. This extended helical structure (pitch of 30 Å) has significant flexibility—the helix encompasses just 25% of the simulation and frequently unwinds to extended chains (5a-2 and 5a-4) as well as C-curve conformations (5a-3 and 5a-5) that are also present in the backbone (e.g., Y-4 and Y-5). Therefore, O-factor V has only a slight impact on the backbone conformation. However, the glucose side chains (colored cyan in Figure 3g,h) are exposed for antibody binding.

Serogroup 5b adds O-factor 7,8 (glucosylation on O-3 of rhamnose A) which, according to our heuristic hypothesis, should have a similar effect as in serogroup 2, increasing the dominance of helices in the 5b O-Ag conformations. This is in fact the case: although the 5b O-Ag has a similar dominant conformational family (Figure 3h, 5b-1, 24%) to 5a, the more minor C-curve conformations in 5a are replaced with helices in 5b (5b-2, 13%; 5b-4, 7%). The chain also has a unique tight hairpin bend conformation (5a-3, 11%) corresponding to the short *r* values adopted early in the 5b *r* time series (Figure 2i).

In summary, conformational analysis suggest refinement of our proposed three broad heuristics for the effects of substitution on the backbone conformation of the *Shigella* O-Ags, as follows. First, substitution at rhamnose C (present in serogroups 2 and 3) restricts the O-Ag chain to predominantly curved conformations, with a larger substituent (e.g., O-4 glucosylation in serogroup 2) having a greater effect than a smaller one (e.g., O-2 O-acetylation in serotype 3). Second, additional substitution on O-3 of rhamnose A (be it O-acetylation or glucosylation: 2a-3Ac, 2b, 3a, 5b) restricts the O-Ag to helical conformations. Third, substitution at O-3 of rhamnose B (O-factor V, serogroup 5) has only a slight effect on conformation, shifting the backbone to somewhat more extended O-Ag conformations. To explain the origin of these general effects, we now investigate the impact of the substitutions on the constituent glycosidic linkages in the *S. flexneri* O-Ag repeating unit.

### 3.4. O-Ag Glycosidic Linkage Conformations

**Figure 5:**
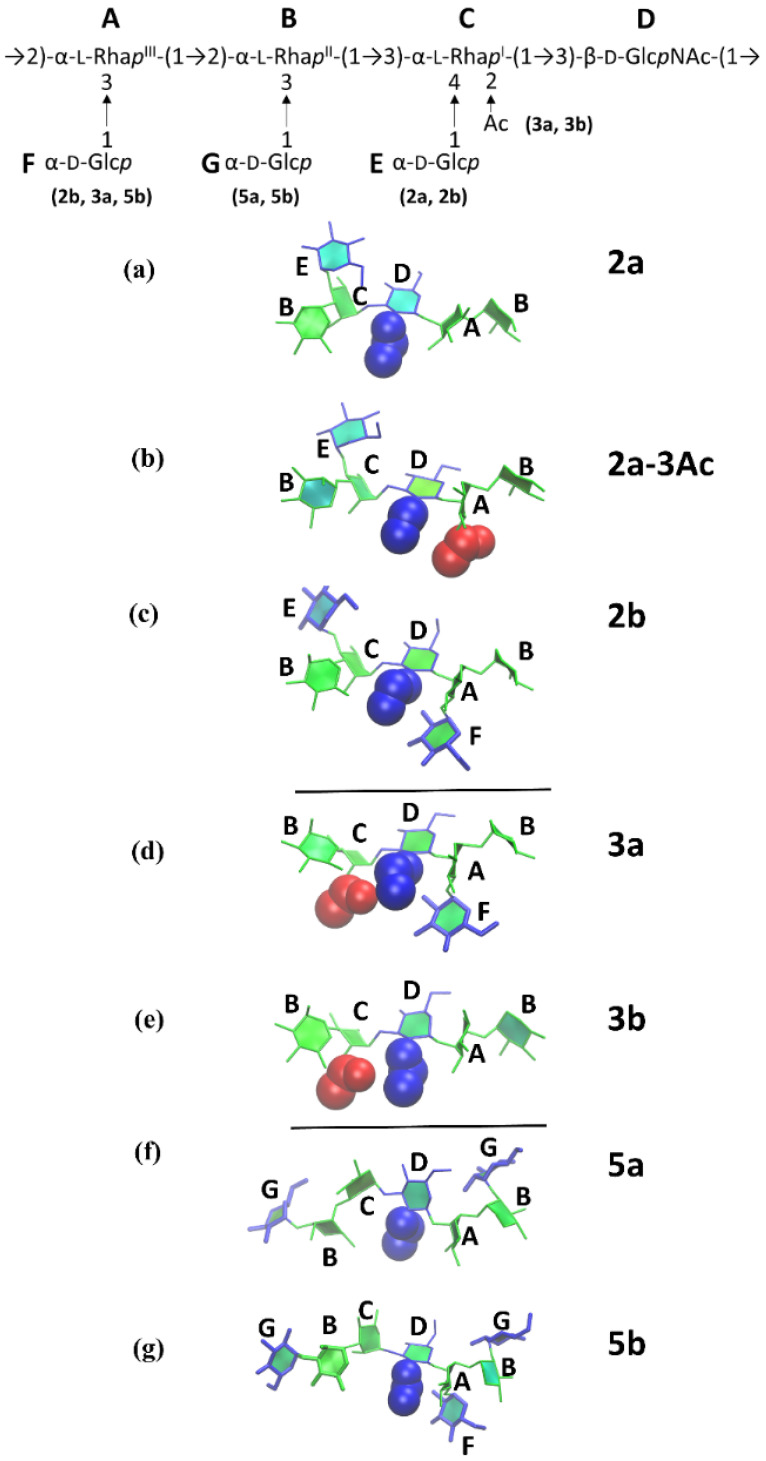
Fragments of the backbone showing the relative arrangements of the sidechains, the N-acetyl (blue) and the O-acetyl (red) substituents for serotypes (**a**) 2a, (**b**) 2a-3Ac, (**c**) 2b, (**d**) 3a, (**e**) 3b, (**f**) 5a, and (**g**) 5b. Glucose rings are colored blue, rhamnose green.

As carbohydrate rings have fairly constrained chair conformations, chain flexibility in the *S. flexneri* O-Ags arises principally from rotations about glycosidic linkages, which are commonly measured via the φ and ψ dihedral angles. Ring substitutions can increase or, more commonly, decrease, the range of motion for a glycosidic linkage. The range of motion for each of the glycosidic linkages in the serogroup 3 and 5 O-Ag RUs over the course of the simulations is shown in Figure 4 with scatter plot heatmaps of the φ, ψ dihedral angle distribution over the course of the simulations. Fragments of the O-Ag backbone showing the relative arrangements of the sidechains, the N-acetyl (blue) and the O-acetyl (red) substituents for each of the serotypes are shown in Figure 5.

For the Y-backbone (Figure 4a), the φ dihedral for all linkages is restricted to a narrow range of values around φ ≈ 40°, whereas the ψ dihedral is more flexible with two primary conformations at ψ ≈ 10° and ψ ≈ −35° (hereafter referred to as +ψ and −ψ, respectively). The D-A linkage is the most constrained, having the narrowest range for psi, because the close proximity of the N-acetyl group to this β-D-Glc*p*NAc-(1→2)-α-L-Rha*p*^III^ linkage restricts ψ rotations. The backbone dihedral angle conformations are consistent with the scatter plots of the φ, ψ linkages from short (60 ns) simulations of 3 RU chains [27]. Further, the estimates of key NOE distances (by r^6^ averaging) are in good agreement with NMR NOE measurements for the native LPSs of serotype 3a [27] (Supplementary Materials Table S1) and 5a [24] (Supplementary Materials Table S2), providing validation for our MD simulations [57].

Comparison of the heatmaps for substituted O-Ags with the backbone (serotype Y) maps allows a closer identification of the specific effect of substitutions on the O-Ag local chain flexibility and dynamics.

For the first heuristic, we found that substitution at rhamnose C (serogroups 2 and 3) restricts the extension of the O-Ag chain to predominantly curved conformations. Comparison of the φ, ψ heatmaps for the Y backbone (Figure 4a) with those for serogroup 2 (Figure 4b–d) shows that the B-C glycosidic linkage (α-L-Rha*p*^II^-(1→3)-α-L-Rha*p*^I^) is considerably restricted in serogroup 2 as compared to the unsubstituted backbone. Serogroup 2 is glucosylated at O-4 of rhamnose C (O-factor II); steric hindrance by this glucose side chain restricts the range of freedom in the neighboring ψ dihedral of the α-(1→3) B-C linkage—see Figure 5a–c. Serogroup 3 (Figure 4e,f) shows a lesser restraint on the B-C linkage, but the α-(1→3) C-D linkage is also restricted relative to the backbone. Serotype 3 is O-2-acetylated on rhamnose C (O-factor 6), and the O-acetyl group is in close proximity to the N-acetyl group of D (β-D-Glc*p*NAc), as shown in Figure 5d,e. Interactions with this neighboring N-acetyl amplify the restrictive effect of the O-acetylation: the C-D linkage for serotype 3b is constrained with partial access to −ψ conformations (mean ≈ −12°) and glucosylation on rhamnose A (O-factor 7,8) further limits the C-D linkage to +ψ orientations for serotype 3a (mean ≈ −6°). It is interesting that a similar restriction in either of these α-L-Rha*p*-(1→3) linkages (B-C and C-D) to −ψ angles has a similar conformational effect for the serogroup 2 and 3 O-Ags, reducing the extension of the O-Ag chain. In contrast, the serogroup 5 O-Ag B-C linkages remain unconstrained, adopting predominantly +ψ orientations (mean ≈ 7°) that result in a greater prevalence of extended structures despite glucosylation on O-3 of rhamnose B (O-factor V). This accounts for the more extended helical conformation in our molecular dynamics simulations as compared to the static model which was built with −ψ B-C orientations [24].

In contrast, the α-L-Rha*p*^III^-(1→2)-α-L-Rha*p*^II^ linkage (A-B) is flexible across all serogroup 2, 3, and 5 O-Ags (Figure 4, first column), significantly contributing to the conformational flexibility of the O-Ags. For serogroup 2, the linkage shifts to favor –ψ orientations (increasing the prevalence of C-curve conformations) while the serogroup 3 A-B linkages remain largely unchanged compared to the backbone (serotype Y). Inspection of the backbone fragments in Figure 5 shows that the glucose substituent on O-4 of rhamnose C is in closer proximity to the A-B linkage than the less bulky O-acetyl substituent at the O-2 position. For serogroup 5, the adjacent glucose side-chain on O-3 of rhamnose B does impose a small steric hindrance to the A-B linkage, possibly contributing to extension of the O-Ag chains in this serogroup.

For our second heuristic, we found that further substitution on O-3 of rhamnose A shifts the O-Ag to extended helical conformations. Comparison of the heatmaps for 2b (Figure 4d), 3a (Figure 4e), and 5b (Figure 4h) with the serotype Y backbone shows that glucosylation at this position (O-factor 7,8) significantly reduces the range of motion for the ψ dihedral of the D-A β-D-Glc*p*NAc-(1→2)-α-L-Rha*p*^I^ linkage from two dominant conformations at ψ ≈ 15° (+ψ) and ψ ≈ −45° (−ψ) to a single dominant conformation at ψ ≈ 12°. In combination with −ψ orientations for the A-B and B-C linkages, this induces a primary helical conformation for serotype 3a. The glucose side-chain (residue F) is in close proximity to the N-acetyl group of the adjacent D residue (β-D-Glc*p*NAc), as shown in Figure 5c,d. Steric clashes between the glucose substituent and the N-acetyl group hinders rotation around the D-A linkage and reduces chain flexibility. The α-D-Glc*p*-(1→3)-α-L-Rha*p*^III^ side chain (F-A, Figure 4, fifth column) is in turn also restricted by the N-acetyl moiety with a single conformation at φ, ψ ≈ −52°, −36°, which is in agreement with NMR measurements for short serotype 3a fragments that predict a −ψ orientation [31].

O-acetylation at this position (O-factor 9, serotype 2a-3Ac, Figure 4c) has a lesser, but similar, restriction on the range of rotation. Steric clashes between these two groups explain the large conformational restriction produced by glucosylation (serotypes 2b and 3a) as well as O-acetylation (2a-3Ac, Figure 5b) on rhamnose A. The conformational effects of this restraint are to increase the incidence of more regular helical structures in these serotypes.

Finally, for the third heuristic, we found that substitution at O-3 of rhamnose B (O-factor V, serogroup 5) has only a slight effect on conformation, shifting the backbone to somewhat more extended O-Ag conformations. Comparison of the heatmaps for serogroup 5a (Figure 4g) with the backbone shows that glucosylation at this position has little effect on the backbone A-B and B-C linkages: the glucose side chains do not interfere with rotations about the bonds (Figure 5f,g).

## 4. Discussion

Our simulations show that *S. flexneri* serogroups 2, 3, and 5 all have very flexible O-Ags. However, substitutions of the backbone residues limit the range and distribution of chain conformations in different ways. Our analysis has suggested three broad heuristics for the effects of substitution on the backbone conformation of the *Shigella* O-Ags: (1) substitution on rhamnose C has the greatest impact on restricting the extension and conformational range of the O-Ags; (2) substitution at O-3 of rhamnose A (2a-3Ac, 2b, 3a, 5b) also has a strong impact, restricting the O-Ags to predominantly helical conformations; (3) substitution at O-3 of rhamnose B (serogroup 5) has only a slight effect on conformation.

Can this conformational analysis give some insight into whether a quadrivalent vaccine containing *S. flexneri* serotypes 2a, 3a (as well as 6 and *S. sonnei*) could provide broad coverage against *S. flexneri* serotypes 3b, 5a, and 5b? The factors that lead to cross-protection between O-Ags are not well understood. However, an assumption that similar O-Ag conformations is a necessary (if not sufficient) criterion for cross-protection between O-Ags seems reasonable. However, immunodominant substitutions that change the binding surface (but perhaps not the conformation) may be confounding factors. On a conformational basis, we postulate that the two substitutions that produce the greatest conformational effects should be represented in the vaccine serotypes. Therefore, the vaccine should contain serotypes with substitutions on rhamnose C (O-factors II and 6) as well as rhamnose A (O-factors 9 and 7,8). Inclusion of an additional serotype with substitution at rhamnose B (O-factor V) seems less likely to be necessary. On the basis of this argument, the 2a-3Ac serotype containing both O-factors II and 9 would seem to be sufficient, whereas 2a (only substituted on rhamnose C) would not.

Cross-protection within serogroup 2 (2a-3Ac and 2b) seems likely due to the similar helical conformations of the serotypes within the group, and cross-protection with the helices in serogroup 3 and serogroup 5 may be possible. However, this conformational argument does not consider serogroup 3′s immunodominant O-2-acetylation on rhamnose C (O-factor 6) [31,32], which is a strong basis for including this serotype. Further reasons to include 3a are the prevalence of 3a infection, the lack of expected cross-protection from 2a [58,59,60], and potential cross-protection by 3a against 2b arising from the shared glucosylation of rhamnose A (O-factor 7,8). Although serotype 3b is conformationally more similar to the non-acetylated 2a chain, the minor C-curve and helical conformations of 3b may allow for partial cross-reactivity from a 2a-3Ac vaccine component. Further, serotypes 3a and 3b share minor conformational families, which may be sufficient to elicit cross-reactivity. Furthermore, serogroup 3a may provide cross-reactivity with other disease-causing serogroups: serotypes 1b and 4b express O-factor 6 and serotype X expresses O-factor 7,8.

Finally, on the basis of conformational similarity, we suggest that the inclusion of serogroup 5 is not necessary in the vaccine, as serotype 5a shares similar helical structures with the 2a-3Ac chain. Further, the partial O-acetylation on O-3 of rhamnose A (O-factor 9) for serotype 5a could provide cross-reactivity with an O-acetylated serotype 2a vaccine, although the exposure of the O-factor V glucosylation for antibody binding may be a confounding factor. In future work, we will investigate the O-Ags of the next most prevalent serogroups identified by the GEMS report—serogroups 6 and 1—to allow for further development of our heuristics for the conformations of the *S. flexneri* O-antigens.

## Supplementary Materials

The following are available online at https://www.mdpi.com/2076-393X/8/4/643/s1, Figure S1: Blocked standard error calculations for determining the extent of simulation convergence, Table S1. Comparison of serotype 3a 1H distances to NMR measurements and previous MD study, Table S2: Comparison of serotype 5a ^1^H distances to NMR measurements and previous MD study.
