# Supplementary material for: Molecular Modeling of the Shigella flexneri Serogroup 3 and 5 O-Antigens and Conformational Relationships for a Vaccine Containing Serotypes 2a and 3a
Source: Vaccines (Basel). 2020 Nov 2;8(4):643. doi: 10.3390/vaccines8040643 (PMC7712985; doi:10.3390/vaccines8040643)
Supplement: Supplementary file 1 [file vaccines-08-00643-s001.pdf]

Simulation Convergence

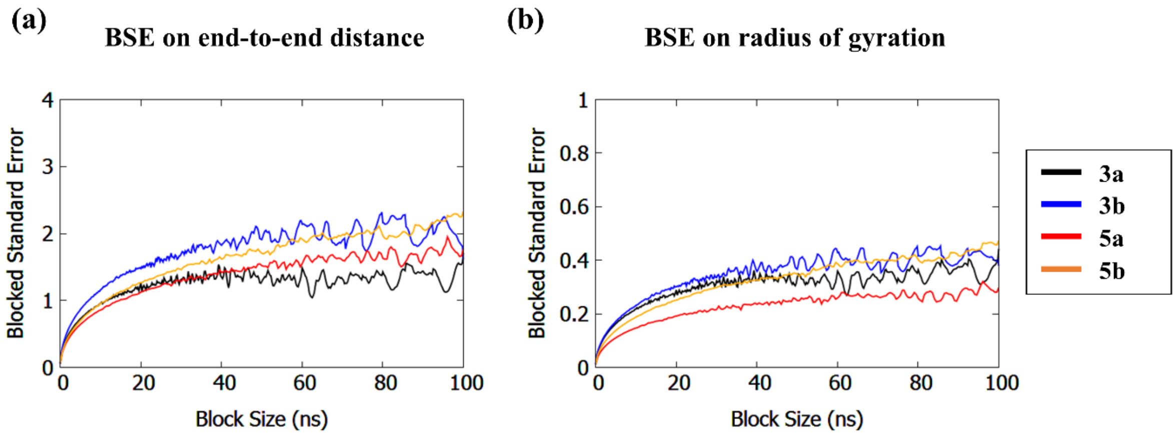

Supplementary Figure S1: Block averaging analysis for the (a) end to end distance, r*, and (b) radius of gyration for 6 RUs of the serogroup 3 and 5 O-Ags. The plot of blocked standard error (BSE) versus block size reaches a plateau for all the O-Ags of serogroup 3 and 5, indicating convergence in each simulation.*

	1A_2B	1A_1D	2A_1D	2A_1F	3A_1F	1B_2C	1B_3C	1C_3D	2C_5B	1D_1F
**Average distance[a]**	**2.3 ± 0.2**	**3.9 ± 0.3**	**2.3 ± 0.2**	**2.3 ± 0.3**	**2.7 ± 0.3**	**3.7 ± 0.6**	**2.3 ± 0.2**	**2.2 ± 0.2**	**2.7 ± 0.4**	**2.8 ± 0.5**
**r^6 average MD[a]**	**2.3 ± 0.2**	**3.8 ± 0.6**	**2.2 ± 0.2**	**2.3 ± 0.2**	**2.6 ± 0.3**	**3.3 ± 0.8**	**2.2 ± 0.2**	**2.2 ± 0.1**	**2.5 ± 0.3**	**2.6 ± 0.4**
**Theillet NMR[b]**	**2.2 ± 0.1**	**3.7 ± 0.4**	**2.3 ± 0.1**	**2.3 ± 0.1**	**2.4 ± 0.2**	**3.3 ± 0.4**	**2.2 ± 0.1**	**2.3 ± 0.1**	**2.7 ± 0.3**	**2.6 ± 0.2**
**Theillet MD[b]**	**2.3**	**3.5**	**2.4**	**2.3**	**2.4**	**3.3**	**2.2**	**2.3**	**2.7**	**2.6**

Supplementary Table S1: Comparison between MD simulation and experimental data of distances between hydrogen atom pairs (columns) are in good agreement for serotype 3a. (a) The mean distance and corresponding r^6 average from our 1.2 µs production runs, excluding 200 ns for equilibration. (b) Experimental NMR distances from NOE build-up curves (estimated 10% error) of the native O-Ag and distances calculated by full ensemble relaxation analysis of 60 ns MD simulations of a 3RU serotype 3a O-Ag (Theillet et. al., 2010).

	1A_1B	1A_2B	1A_4B	1A_1D	1A_2D	2A_1B	2A_1D	2A_2D	2A_5D	2A_1G	4A_1D	5A_1B	5A_2B	1B_2C	1B_3C	1B_4C	2B_4C	2B_1G	3B_1G	3B_5G
**Average distance[a]**	**3.6 ± 0.6**	**2.3 ± 0.2**	**4.2 ± 0.4**	**3.6 ± 0.7**	**4.4 ± 0.3**	**5.0 ± 0.2**	**2.4 ± 0.2**	**4.3 ± 0.2**	**4.0 ± 0.4**	**4.5 ± 0.8**	**4.2 ± 0.4**	**2.6 ± 0.4**	**3.7 ± 0.5**	**4.0 ± 0.4**	**2.3 ± 0.2**	**3.9 ± 0.3**	**4.2 ± 0.3**	**2.4 ± 0.5**	**2.7 ± 0.3**	**3.0 ± 0.6**
**r^6 average[a]**	**3.2 ± 0.8**	**2.2 ± 0.2**	**4.0 ± 1.1**	**3.1 ± 0.8**	**4.3 ± 0.4**	**4.9 ± 0.2**	**2.3 ± 0.2**	**4.3 ± 0.8**	**3.9 ± 0.4**	**3.9 ± 1.4**	**4.0 ± 0.8**	**2.4 ± 0.3**	**3.4 ± 0.9**	**3.6 ± 1.5**	**2.3 ± 0.2**	**3.7 ± 1.1**	**4.1 ± 0.4**	**2.3 ± 0.2**	**2.6 ± 0.4**	**2.7 ± 0.4**
**ROE[b]**	**3.1 ± 0.3**	**2.1 ± 0.2**	**4.7 ± 0.5**		**4.5 ± 0.4**		**2.0 ± 0.2**	**4.5 ± 0.4**	**4.1 ± 0.4**	**4.1 ± 0.4**	**4.2 ± 0.4**	**2.4 ± 0.2**	**4.6 ± 0.5**	**3.4 ± 0.3**	**2.1 ± 0.2**	**4.3 ± 0.4**	**4.6 ± 0.5**	**2.3 ± 0.2**	**2.5 ± 0.2**	**3.0 ± 0.3**
**NOE[b]**	**3.0 ± 0.3**	**2.1 ± 0.2**	**4.6 ± 0.5**	**3.5 ± 0.3**		**4.7 ± 0.5**	**2.1 ± 0.2**	**4.5 ± 0.4**	**3.8 ± 0.4**	**4.1 ± 0.4**	**4.3 ± 0.4**	**2.6 ± 0.3**	**4.3 ± 0.4**	**2.9 ± 0.3**	**2.1 ± 0.2**	**4.4 ± 0.4**	**4.8 ± 0.5**	**2.4 ± 0.2**	**2.2 ± 0.2**	
**Theillet MD[c]**	**3.2**	**2.3**	**4.5**	**3.2**		**4.9**	**2.3**	**4.5**	**4.1**	**4.0**	**4.2**	**2.4**	**3.9**	**3.3**	**2.3**	**4.0**	**4.4**	**2.3**	**2.6**	

Supplementary Table S2: Distances of pairs of hydrogen atoms (columns) from MD simulation and experimental data for serotype 5a. (a) The average distance and corresponding r^6 average from our 2 µs production runs, excluding 200 ns for equilibration. (b) Experimental NMR distances (estimated 10% error) derived from NOE build-up curves and ROESY off-resonance measurements on the native O-Ag sample with an avDP15 (Clement et. al., 2003). (c) Distances calculated by full ensemble relaxation analysis of 60 ns MD simulations of a 3RU O-Ag (Theillet et. al., 2010).
